# Calcium Oxalate Induces Renal Injury through Calcium-Sensing Receptor

**DOI:** 10.1155/2016/5203801

**Published:** 2016-11-14

**Authors:** Xiaoran Li, Junhai Ma, Wei Shi, Yu Su, Xu Fu, Yanlin Yang, Jianzhong Lu, Zhongjin Yue

**Affiliations:** ^1^Department of Urology, Institute of Urology, Gansu Nephro-Urological Clinical Center, Key Laboratory of Urological Diseases in Gansu Province, The Second Hospital of Lanzhou University, Lanzhou, Gansu 730030, China; ^2^Department of Urology, The First Affiliated Hospital of Anhui Medical University and Institute of Urology, Anhui Medical University, Hefei, China; ^3^The First Hospital of Lanzhou University, Lanzhou, China

## Abstract

*Objective.* To investigate whether calcium-sensing receptor (CaSR) plays a role in calcium-oxalate-induced renal injury.* Materials and Methods.* HK-2 cells and rats were treated with calcium oxalate (CaOx) crystals with or without pretreatment with the CaSR-specific agonist gadolinium chloride (GdCl_3_) or the CaSR-specific antagonist NPS2390. Changes in oxidative stress (OS) in HK-2 cells and rat kidneys were assessed. In addition, CaSR, extracellular signal-regulated protein kinase (ERK), c-Jun N-terminal protein kinase (JNK), and p38 expression was determined. Further, crystal adhesion assay was performed* in vitro*, and the serum urea and creatinine levels and crystal deposition in the kidneys were also examined.* Results.* CaOx increased CaSR, ERK, JNK, and p38 protein expression and OS* in vitro* and* in vivo*. These deleterious changes were further enhanced upon pretreatment with the CaSR agonist GdCl_3_ but were attenuated by the specific CaSR inhibitor NPS2390 compared with CaOx treatment alone. Pretreatment with GdCl_3_ further increased* in vitro* and* in vivo* crystal adhesion and renal hypofunction. In contrast, pretreatment with NPS2390 decreased* in vitro* and* in vivo* crystal adhesion and renal hypofunction.* Conclusions.* CaOx-induced renal injury is related to CaSR-mediated OS and increased mitogen-activated protein kinase (MAPK) signaling, which subsequently leads to CaOx crystal adhesion.

## 1. Introduction

Urolithiasis is the third most common disease of the urinary tract, and it respects no geographical, racial, or cultural boundaries. Further, it is associated with a high recurrence rate [1]. Stones composed of calcium oxalate (CaOx), either alone or mixed with calcium phosphate, are hitherto the most common uroliths, accounting for more than 80% of stones [2]. Although currently available stone removal techniques, such as extracorporeal shock wave lithotripsy (ESWL), ureteroscopy (URS), and percutaneous nephrolithotomy (PNL), are considered effective, they are costly. Thus, these techniques are an option for only a limited number of patients [3]. Additionally, compelling data suggest that these techniques have some serious side effects [3]. Despite the substantial progress of studies of the biological and physical manifestations of urolithiasis, the mechanism underlying CaOx stone formation remains unclear. In our previous study, we found that apocynin, “a selective NADPH oxidase inhibitor,” reduces crystal deposition by modulating oxidative stress (OS) and thereby decreasing urolithiasis-related protein expression [4].

Calcium-sensing receptor (CaSR), a member of the G-protein-coupled receptor (GPCRs) superfamily, plays significant roles in cell hormone secretion, inflammation, apoptosis, and differentiation [5–7]. CaSR is organized into three major structural domains: a large amino-terminal extracellular domain, the typical seven transmembrane domains, and a cytoplasmic carboxy terminus [8]. It is modulated by not only Ca^2+^ but also a large number of ions/molecules, including many cations (e.g., Gd^3+^ and Mg^2+^), polyamines, and some antibiotics [9]. In different populations, a polymorphism in the CaSR gene leading to the Arg990Gly mutation has been reported to be associated with hypercalciuria and nephrolithiasis [10].

CaOx increases OS, which regulates crystal formation, growth, and retention through the timely production of crystallization modulators [11]. OS upregulates the expression of various factors, including bikunin, osteopontin (OPN), and the Tamm-Horsfall protein, and activates the p38 mitogen-activated protein kinase (MAPK) pathway. MAPKs, an important group of serine and threonine signaling kinases, consist of the following three family members: (1) c-Jun N-terminal kinase (JNK) MAPK, (2) p38 MAPK, and (3) ERK MAPK. Koul et al. [12] have reported that the p38 MAPK signaling pathway is activated by calcium oxalate monohydrate (COM) crystals and that this pathway may transduce critical signals for DNA synthesis in renal epithelial cells [12].

CaOx-induced OS, which causes further injury to renal cells, is considered a risk factor for crystallization and crystal deposition in the kidneys because it promotes crystal nucleation, aggregation, and retention and stone development [13, 14]. Wang et al. [15] have reported that lipopolysaccharide-induced cardiomyocyte injury is related to CaSR-mediated OS and increases in the tumor necrosis factor-*α* (TNF-*α*) and interleukin-6 (IL-6) levels. It remains unknown whether CaSR plays a role in CaOx-induced injury in cells and in animal models of urolithiasis.

Therefore, the present study was designed to examine the possible effects of CaSR in a model of CaOx-induced nephrolithiasis and to provide direct physiological and biochemical evidence of whether CaSR is involved in this process.

## 2. Materials and Methods

### 2.1. Materials

Quinoxaline-2-carboxylic acid adamantan-1-ylamide (NPS2390, CAS number 226878-01-9) was purchased from Tocris Bioscience (USA). GdCl_3_ (product number 43977-0) was purchased from Sigma-Aldrich (St. Louis, MO, USA). Anti-phospho-p38 (ab45381), anti-phospho-JNK (ab4821), anti-phospho-ERK (ab21396), and anti-CaSR (ab79038) antibodies were purchased from Abcam (USA). Glyceraldehyde-3-phosphate dehydrogenase (GAPDH) was purchased from Sigma-Aldrich (St. Louis, MO, USA).

### 2.2. Preparation of COM Crystals

COM crystals were prepared as previously described [16]. Briefly, equal volumes of 10 mM sodium oxalate and 10 mM CaCl_2_ were mixed at room temperature. COM crystals formed immediately, and the suspension was equilibrated for 3 days at 4°C. The crystals were then washed with deionized water and dried at 60°C. The crystals were confirmed to be COM by Fourier transform infrared spectroscopy. A stock solution of COM (5 mg/mL) was prepared in sterile phosphate-buffered saline (PBS). The COM crystal solution was distributed homogeneously across the monolayer, where it settled on top of the cells under the force of gravity (*μ*g/cm^2^ of cells). In addition, in our experiment, when the COM stock solution was added to the cells, PBS was also added so that the volumes were the same for all groups.

### 2.3. Cell Culture and Treatment

HK-2 cells were purchased from the National Platform of Experimental Cell Resources for Science (Beijing, China). Human kidney epithelial cells (HK-2) were maintained in Dulbecco's modified Eagle's medium (DMEM) supplemented with 10% fetal bovine serum (FBS) and 1% antibiotics. The cultures were maintained at 37°C in a 5% CO_2_/95% O_2_ humidified atmosphere. COM was prepared using the above-described method. HK-2 cells were stimulated with COM at different doses (0, 33.5, 67, 134, or 268 *μ*g/cm^2^ of cells) for different periods of time (3 to 48 h) to determine changes in CaSR expression* in vitro*. Then, CaSR protein expression levels were measured by western blotting. HK-2 cells were pretreated with the CaSR activator GdCl_3_ (300 *μ*M) for 30 min or with the CaSR inhibitor NPS2390 (10 *μ*M) for 60 min and were then exposed to COM (67 *μ*g/cm^2^ of cells) for 6 h. The medium was removed and saved, and the cells were washed three times with serum-free DMEM and harvested for further analysis. Then, the cells were collected, and the levels of superoxide dismutase (SOD), malondialdehyde (MDA), CaSR, and MAPK were determined. The medium was removed and saved for measurement of the 8-isoprostane (8-IP) level.

### 2.4. COM Crystal Adhesion Assay

COM crystal adhesion assay was performed using a previously described method [17]. Three days after HK-2 cells were seeded and cultured, they were randomly divided into the following four groups: (1) control group: HK-2 cells were continuously cultured at 37°C for 6 h in DMEM; (2) COM group: HK-2 cells were incubated with COM (67 *μ*g/cm^2^ of cells) at 37°C for 6 h; (3) COM+GdCl_3_ group: HK-2 cells were pretreated with the CaSR activator GdCl_3_ (300 *μ*M) for 30 min, and then COM crystals were added to the growth medium (67 *μ*g crystals/cm^2^ of cells) at 37°C for 6 h; and (4) COM+NPS2390 group: HK-2 cells were pretreated with the CaSR inhibitor NPS2390 (10 *μ*M) for 60 min, and then COM crystals were added to the growth medium (67 *μ*g crystals/cm^2^ of cells) at 37°C for 6 h.

Next, the unbound crystals were removed, and the cells were washed three times with PBS. The remaining crystals adhered on the cell surface were counted in 15 randomized high-power fields (HPFs) under a phase contrast microscope (Olympus CKX41, Olympus Co. Ltd., Tokyo, Japan). This experiment was performed in triplicate for all conditions.

### 2.5. Experimental Animals and Animal Treatments

Sprague-Dawley (SD) rats (180 to 200 g) from the Animal Experiment Center of Lanzhou University were maintained under standard laboratory conditions (25 ± 2°C at 40 to 60% relative humidity with a 12-hour light-dark cycle). All of the procedures and experimental protocols were approved by the Institutional Animal Care and Use Committee of Lanzhou University. Forty male SD rats were divided into 4 equal groups. The control group was fed a standard diet and served as the intact control group. The ethylene glycol (EG) group was fed a standard commercial diet with 1% EG for 28 days to induce urolithiasis. The EG+GdCl_3_ group received EG and was injected with 8.67 mg/kg/day GdCl_3_ for 4 weeks. The EG+NPS-2390 group received EG and was injected with 0.20 g/kg NPS-2390 for 4 weeks [8]. After 4 weeks, the rats were euthanized, and the left kidneys were fixed with formalin for hematoxylin and eosin (H&E) staining. The right kidneys were maintained in a −80°C freezer for western blotting.

### 2.6. Biochemical Examination

The serum creatinine and blood urea nitrogen levels were determined using an automatic clinical chemistry analyzer (Hitachi 7600, Tokyo, Japan).

### 2.7. OS Studies

Cells were lysed using a cell lysis buffer at room temperature, and, after approximately 20 min, lysates were collected for measurement of the SOD and MDA levels. Nine volumes of cold phosphate buffer (pH 7.4) were mixed with 0.1 g kidney tissues, and the tissues were then homogenized. Next, the homogenates were centrifuged (16,000 ×g at 4°C for 5 min) to obtain supernatants. Both the supernatants and cell lysates were used for measurement of the SOD and MDA levels using two kits (Jiancheng Bioengineering, Nanjing, China). In addition, the urine and media were collected for measurement of the 8-IP levels using an enzyme-linked immunosorbent assay kit, according to the manufacturer's instructions (Quantikine Assay, R&D Systems, USA).

### 2.8. Kidney Crystal Deposition

Paraffin-embedded kidney sections (5 *μ*m) were stained with H&E. The number of crystal depositions in the renal tubules was determined by assessing 10 randomly selected fields per kidney. Crystal deposition scoring included the following scores: (1) no deposition = 0 points; (2) deposition in the papillary tip = 1 point; (2) deposition in the corticomedullary junction = 2 points; and (3) deposition in the cortex = 3 points. If crystal depositions were observed at multiple sites, the points were combined for a total score per kidney.

### 2.9. Pathological Examination

The pathological changes were scored according to the area of renal injury as follows: (1) invisible lesions = 0 points; (2) tubule interstitial inflammatory infiltration with a lesion area of <20% and mild dilation of tubules = 1 point; (3) tubule interstitial inflammatory infiltration with a lesion area of <40% and obvious dilation of tubules = 2 points; and (4) tubule interstitial inflammatory infiltration with a lesion area of >40% and severe dilation of tubules = 3 points.

### 2.10. Western Blotting Analysis

A total of 0.1 g renal tissue in 1 mL lysis buffer mixed with 10 *μ*L phenylmethanesulfonyl fluoride (PMSF, 100 mM) and NaF (100 mM) was homogenized. Then, the homogenates were centrifuged (12,000 ×g for 5 min) at 4°C, and the supernatants were collected. After being washed twice with ice-cold PBS, the cells were incubated with protein lysates containing PMSF and NaF for 30 min. Then, the cells were collected, mechanically disrupted, and centrifuged at 12,000 rpm for 15 min at 4°C to isolate the nuclei. A BCA protein assay kit was used to determine the protein concentrations. Equivalent amounts of protein were mixed with SDS-PAGE sample loading buffer. The mixture was heated at 100°C for 5 min. Then, the samples were run via sodium dodecyl sulfate-polyacrylamide gel electrophoresis (SDS-PAGE) using a 10% acrylamide resolving gel, and the separated proteins were transferred to polyvinylidene fluoride (PVDF) membranes. The membranes were blocked with 5% skim milk powder at room temperature for 2 h. Then, they were incubated with a phospho-JNK, phospho-p38, or phospho-ERK antibody at 4°C overnight. Subsequently, they were washed three times in TBST for 10 min, incubated with the respective antibody labeled with horseradish peroxidase (1 : 5000) for 2 h, and washed again with TBST as described above. Finally, the proteins were detected by enhanced chemiluminescence.

### 2.11. Statistical Analysis

The data are presented as the mean ± standard error. Student's *t*-test for comparisons between two groups and one-way analysis of variance (ANOVA) for comparisons between three or more groups were used to detect significant differences. A *P* value of <0.05 was considered statistically significant.

## 3. Results

CaSR protein expression in HK-2 cells exposed to varying COM doses (0, 33.5, 67, 134, or 268 *μ*g/cm^2^ of cells) for varying amounts of time (3 to 48 h) was determined by western blotting.

**Figure 1:**
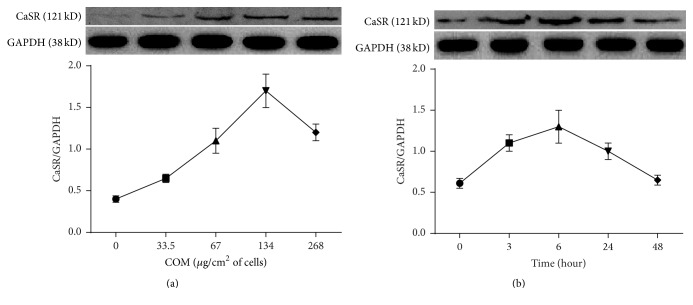
COM affects CaSR protein expression in HK-2 cells. (a) Cells were induced by COM (0, 33.5, 67, 134, or 268 *μ*g/cm^2^ of cells) for 6 h, and then CaSR protein expression was examined by western blotting. (b) Cells were treated with COM (67 *μ*g/cm^2^ of cells) for 0, 3, 6, 24, and 48 h, and then CaSR protein expression was examined by western blotting. The data are presented as the mean ± standard error of the mean (SEM) (*n* = 5).

The response was both concentration- and time-dependent (Figures 1(a) and 1(b)).  Figure 1(a) indicates that CaSR protein expression in HK-2 cells was the highest following exposure to 134 *μ*g COM crystals/cm^2^ of cells.  Figure 1(b) indicates that CaSR protein expression was the highest following exposure to 67 *μ*g crystals/cm^2^ of cells for 6 h.

### 3.1. Effects of CaSR on P-ERK1/2, P-JNK, and P-p38 Protein Expression in HK-2 Cells Exposed to COM

**Figure 2:**
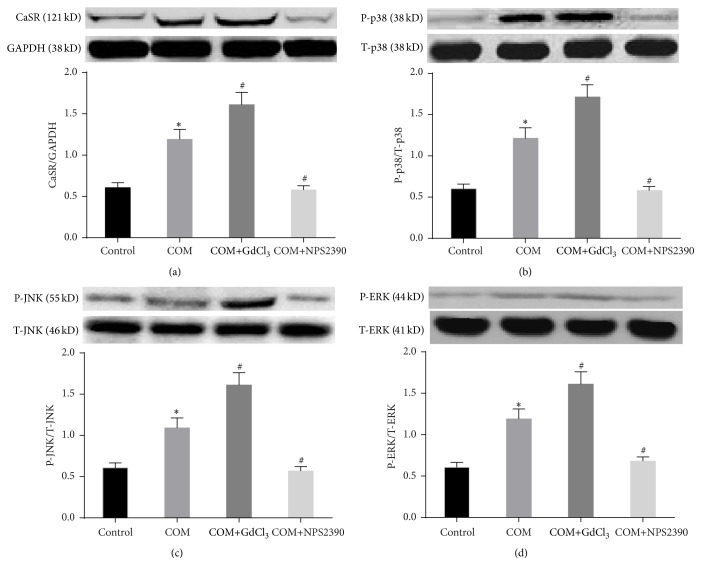
CaSR and MAPK expression in HK-2 cells in the different groups, as determined by western blotting. (1) Control group: HK-2 cells were continuously cultured at 37°C for 6 h in DMEM. (2) COM group: HK-2 cells were incubated with COM (67 *μ*g/cm^2^ of cells) at 37°C for 6 h. (3) COM+GdCl_3_ group: HK-2 cells were pretreated with the CaSR activator GdCl_3_ (300 *μ*M) for 30 min, and then COM crystals were added to the growth medium (67 *μ*g crystals/cm^2^ of cells) at 37°C for 6 h. (4) COM+NPS2390 group: HK-2 cells were pretreated with the CaSR inhibitor NPS2390 (10 *μ*M) for 60 min, and then COM crystals were added to the growth medium (67 *μ*g crystals/cm^2^ of cells) at 37°C for 6 h. The data are expressed as the mean ± SEM. ^*∗*^ *P* < 0.05 compared with the control group and ^#^ *P* < 0.05 compared with the COM group (*n* = 5).

To determine whether CaSR is involved in the mitogen-activated protein kinase signaling pathway, ERK1/2, JNK, and p38 expression was analyzed by western blotting. The results showed that the total ERK1/2, JNK, and p38 expression levels were similar among the different groups. Following exposure of the cells to COM, CaSR, P-p38, P-ERK1/2, and P-JNK expression was upregulated (Figure 2) (*P* < 0.05), and this effect was further enhanced by GdCl_3_ and reduced by NPS-2390 (*P* < 0.05) (Figures 2(a), 2(b), 2(c), and 2(d)).

### 3.2. Effects of CaSR on COM Adherence to Cells in Crystal Adhesion Assay

**Figure 3:**
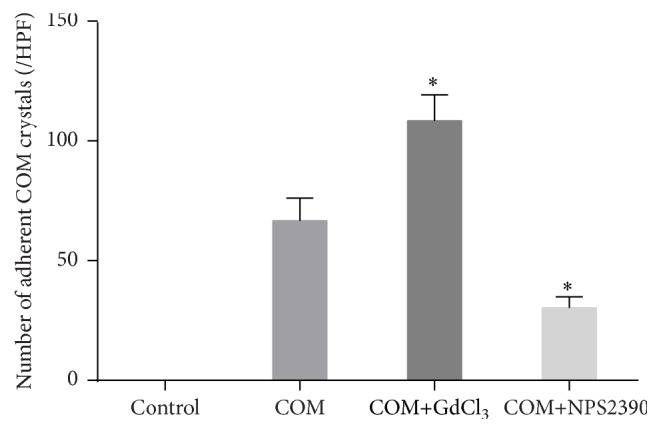
COM crystal adhesion assay. (1) Control group: HK-2 cells were continuously cultured at 37°C for 6 h in DMEM. (2) COM group: HK-2 cells were incubated with COM (67 *μ*g/cm^2^ of cells) at 37°C for 6 h. (3) COM+GdCl_3_ group: HK-2 cells were pretreated with the CaSR activator GdCl_3_ (300 *μ*M) for 30 min, and then COM crystals were added to the growth medium (67 *μ*g crystals/cm^2^ of cells) at 37°C for 6 h. (4) COM+NPS2390 group: HK-2 cells were pretreated with the CaSR inhibitor NPS2390 (10 *μ*M) for 60 min, and then COM crystals were added to the growth medium (67 *μ*g crystals/cm^2^ of cells) at 37°C for 6 h. After removing the unbound crystals, the remaining crystals adhered on the cell surface were counted in 15 randomized high-power fields (HPFs) under a phase contrast microscope (Olympus CKX41). Each bar was generated from 3 independent experiments. ^*∗*^ *P* < 0.05 compared with the COM group.

We also evaluated the functional significance of the increase in CaSR expression induced by COM. We hypothesized that high CaSR expression could enhance COM crystal adhesion to the cell surface, thus playing a significant role in this phenomenon. Our hypothesis was addressed by performing COM crystal adhesion assay. The results demonstrated that GdCl_3_ significantly increased the number of adherent COM crystals (Figure 3) (*P* < 0.05). Moreover, neutralization of CaSR expression by NPS-2390 resulted in a dramatic reduction in the number of adherent COM crystals (*P* < 0.05).

### 3.3. Changes in SOD, MDA, and 8-IP Levels in the Four Groups of HK-2 Cells

**Figure 4:**
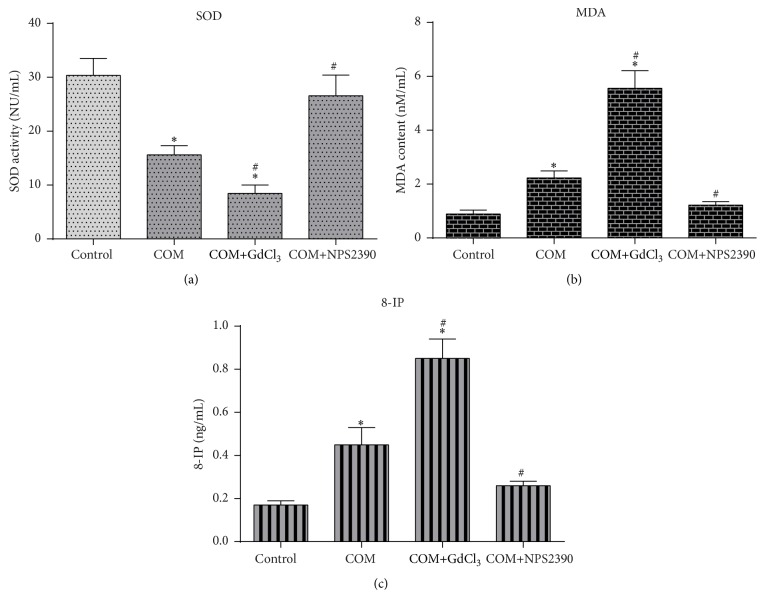
Levels of SOD activity and MDA in cells and of 8-IP in culture medium in the different groups. ^*∗*^ *P* < 0.05 compared with the control group and ^#^ *P* < 0.05 compared with the COM group (*n* = 10).

The MDA and 8-IP levels were increased and the SOD level was decreased in COM-treated HK-2 cells compared with untreated cells (*P* < 0.05) (Figures 4(a), 4(b), and 4(c)). OS was further increased after treatment with GdCl_3_. However, when CaSR expression was decreased by NPS-2390, SOD activity was restored, and the MDA and 8-IP levels were reduced compared with those in the COM-treated cells (Figures 4(a), 4(b), and 4(c)) (*P* < 0.05).

### 3.4. Serum Urea, Serum Creatinine, and OS in Rats

As shown in Table 1, increases in the serum urea and serum creatinine levels were observed in the EG group compared with the control group, and treatment with NPS2390 improved renal function to approximately normal levels. However, treatment with GdCl_3_ further decreased renal function compared with that in the EG group (*P* < 0.05).

The MDA and 8-IP levels were significantly elevated in the EG group, while SOD activity was decreased, indicating that CaOx induced OS. In addition, the MDA and 8-IP levels were decreased and SOD activity was restored in the EG+NPS2390 group compared with the EG group (*P* < 0.05). However, the levels of these OS indicators were significantly elevated in the EG+GdCl_3_ group compared with the EG group (*P* < 0.05).

### 3.5. CaOx Crystal Deposition and Histological Examination

**Figure 5:**
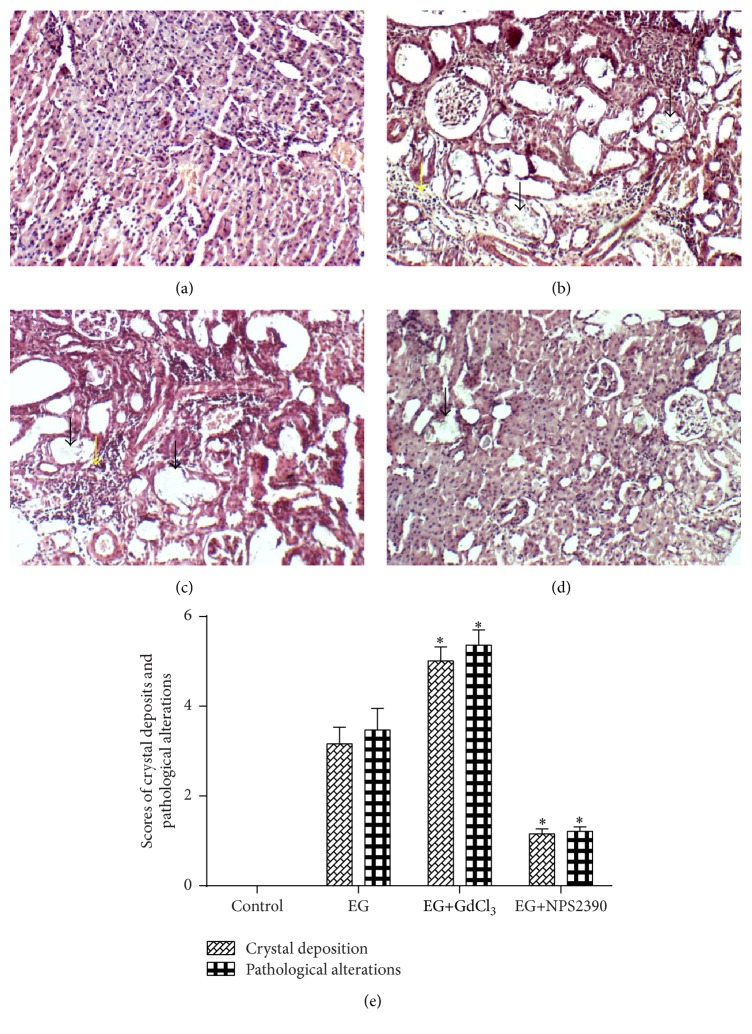
Representative microscopic photographs showing crystal deposits and pathological changes in rat kidneys. (a) The control group rats were fed a standard diet and served as the intact control group. (b) The EG group rats were fed a standard commercial diet with 1% ethylene glycol for 28 days to induce nephrolithiasis. (c) The EG+GdCl_3_ group rats received EG and were injected with 8.67 mg/kg/day GdCl_3_ for 4 weeks. (d) The EG+NPS2390 group rats received EG and were injected with 0.20 g/kg NPS2390 for 4 weeks. (e) Scores for crystal deposition and pathological alterations. The columns and bars represent the mean ± SEM (^*∗*^ *P* < 0.05 compared with the EG group, magnification: ×100, *n* = 10).

The H&E-stained kidney sections were observed using an optical microscope (Figures 5(a), 5(b), 5(c), and 5(d)). CaOx crystals (black arrow) were noted in all parts of the kidney in the EG group (Figure 5(b)), especially in the renal cortex. Few crystals were observed in the EG+NPS2390 group compared with the EG group (*P* < 0.05) (Figure 5(e)). The scoring of crystal deposition revealed a significantly increased number of deposits in the EG+GdCl_3_ group compared with the EG group (Figure 5(e)). Histological examination revealed that the EG group exhibited severe tubule dilation and inflammatory infiltration (yellow arrow) (Figure 5(b)). The pathological score of the EG+NPS2390 group was significantly reduced compared with that of the EG group (*P* < 0.05) (Figure 5(e)); however, the pathological score of the EG+GdCl_3_ group was increased (*P* < 0.05) (Figure 5(e)).

### 3.6. Western Blot Analysis of CaSR in the Kidneys

**Figure 6:**
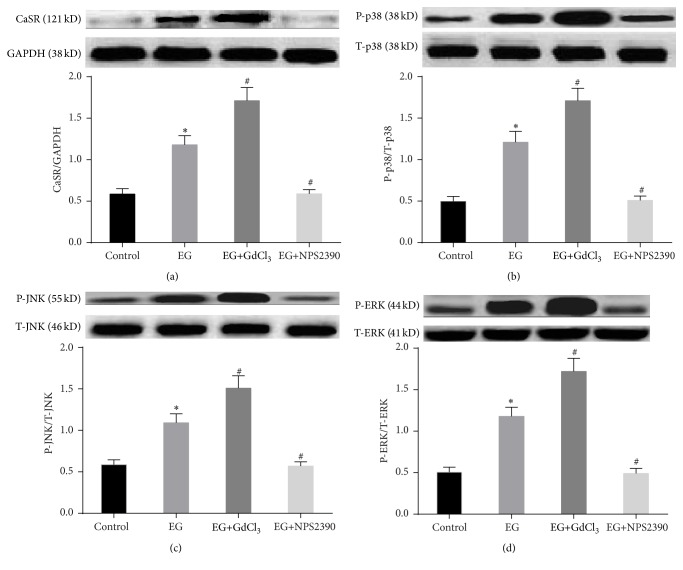
Detection of CaSR and MAPK protein expression in rat kidneys in the different groups. Western blot analysis of CaSR (a) expression in rat kidneys. Western blot analysis of phosphorylated p38 (b), JNK (c), and ERK1/2 (d) expression and of total p38, JNK, and ERK1/2 expression in rat kidneys. The data are expressed as the mean ± SEM. ^*∗*^ *P* < 0.05 compared with the control group and ^#^ *P* < 0.05 compared with the EG group (*n* = 5).

CaSR expression was observed in all of the groups (Figure 6(a)). However, its expression was weak in the control group. In contrast, western blot analysis revealed significant upregulation of CaSR expression in the EG group (Figure 6(a)). In addition, its expression was increased in the EG+GdCl_3_ group compared with the EG group (*P* < 0.05) (Figure 6(a)) and was reduced in the EG+NPS2390 group (*P* < 0.05) (Figure 6(a)).

### 3.7. Effects of CaSR on ERK1/2, JNK, and p38 Protein Expression in Nephrolithiasis Rats

The results showed that the total ERK1/2, JNK, and p38 protein levels were similar among the different groups. Following treatment of the rats with EG, P-ERK1/2, P-p38, and P-JNK expression was upregulated (*P* < 0.05). In addition, when the high CaSR expression in the EG group was silenced by NPS2390, P-ERK1/2, P-p38, and P-JNK expression was decreased (*P* < 0.05) (Figures 6(b), 6(c), and 6(d)). Moreover, when the already high CaSR expression in the EG group was further enhanced by GdCl_3_ treatment, P-ERK1/2, P-p38, and P-JNK expression was also increased (*P* < 0.05) (Figures 6(b), 6(c), and 6(d)).

## 4. Discussion

CaSR is expressed in renal tissues. Increasing evidence indicates that CaSR may play a role in the formation of kidney stones. However, its role in CaOx-induced renal dysfunction has yet to be fully elucidated. CaOx-induced renal injury causes structural changes to the kidney; in addition to decreased renal function, changes in renal injury signaling pathways, renal protein levels, apoptosis, and fibrosis have been reported [18, 19]. Studies have also demonstrated that renal epithelial cells respond to increased CaOx crystal formation by producing macromolecular proteins, such as p38, OPN, hyaluronic acid, a-1-microglobulin, Tamm-Horsfall protein, and prothrombin fragment-1, which subsequently affect the crystal deposition process [12, 20].

CaSR, the encoding gene of which is a candidate gene for nephrolithiasis, may underlie the predisposition of some individuals to calcium nephrolithiasis [21]. Studies have demonstrated that injury to the rat myocardium induced by lipopolysaccharide is mediated by CaSR [15]. However, little is known about the role of CaSR in the formation of CaOx nephrolithiasis. In the present study, we used NPS2390 (a specific CaSR antagonist) and GdCl_3_ (a specific CaSR activator) to evaluate the involvement of CaSR in CaOx nephrolithiasis and to explore the possible underlying mechanism.

The results showed that EG administration for 28 days led to an increase in CaSR protein expression in the kidneys of the lithic model rats. These data strongly indicate that CaSR is involved in nephrolithiasis formation. The results of the experiment using HK-2 cells further confirmed this phenomenon. COM treatment resulted in a time- and concentration-dependent increase in CaSR expression in HK-2 cells. Although many cations (e.g., Gd^3+^ and Mg^2+^), polyamines, and some antibiotics activate CaSR expression [9], we found that CaOx crystals also induced its expression in renal epithelial cells in both the* in vitro* experiments and animal models in the present study. In contrast with many cations (e.g., Gd^3+^ and Mg^2+^), COM is an insoluble crystal and cannot penetrate cells. Thus, we suggest that COM may activate CaSR through its large amino terminus in the extracellular domain. However, further experiments are needed to verify this hypothesis.

High OS, activation of MAPK signaling pathways, renal dysfunction, and massive CaOx deposition were also observed in HK-2 cells and renal tubules compared with those in the control groups. Furthermore, these changes were enhanced or weakened by a CaSR agonist or its antagonist, respectively, suggesting that CaSR activation regulates these indexes.

MAPKs [22, 23], an important group of serine and threonine signaling kinases, consist of the following three family members: (1) JNK MAPK, (2) p38 MAPK, and (3) ERK MAPK [24]. A recent study has demonstrated that CaSR upregulates the p38 MAPK pathway in H9c2 cardiomyoblast cells [25]. Additionally, Koul et al. [12] have reported that the p38 and JNK signal transduction pathways are activated by COM crystals in renal epithelial cells [12]. We used a CaSR antagonist and activator to evaluate the relationship between MAPK and CaSR in the formation of nephrolithiasis. We found that COM increased the P-38, P-ERK, and P-JNK levels, consistent with the results of previous studies [12, 18, 19]. These increases could be enhanced by GdCl_3_ or reduced by NPS2390 compared with the COM group, suggesting that CaSR mediates the COM-induced activation of the MAPK signaling pathway in HK-2 cells. Clearly, the activation of MAPK is involved in many complex processes, including cell death inhibition and the production of inflammatory cytokines following cellular stress [26]. JNK mediates various cellular responses, including programmed cell death (apoptosis), epithelial sheet movement, and planar polarity [27]. Additionally, cellular death may lead to adherence of an increased number of crystals to the surfaces of HK-2 cells, as well as increased crystal formation. Studies have also suggested that p38 MAPK activation by OS, pathogens, and proinflammatory cytokines downregulates tight junctions in many cell types [28–30]. The downregulation of tight junctions, which have a role in epithelial barrier function, may lead to increased crystal retention.

The COM-induced generation of OS may be the initial trigger of a vicious cycle of kidney stone formation [31, 32]. An excessive amount of crystals can trigger the production of reactive oxygen species (ROS) in kidney tissues. ROS production leads to renal epithelial injury and apoptosis, which provide sites for crystal attachment and the eventual retention of crystals within the kidneys [33, 34]. Furthermore, cell degradation following renal epithelial injury results in the generation of numerous membranous vesicles, which are effective crystal nucleators, the enhancement of crystal nucleation at low supersaturation, and the promotion of crystal-cell interactions [35]. In our previous study, we have shown that melamine-related nephrolithiasis results in increased OS* in vitro* and* in vivo*. In addition, we have demonstrated that prophylactic apocynin (a selective NADPH oxidase inhibitor) treatment reduces renal melamine-related crystal deposition, potentially by modulating OS and thereby decreasing P-p38 and osteopontin expression. Thus, NADPH oxidase appears to be a particularly significant enzyme among the enzymatic systems involved in melamine-induced OS production [4]. In the present study, we found that OS was significantly increased by exposure to COM crystals. This increased OS could be enhanced by the CaSR agonist GdCl_3_ or attenuated by the CaSR inhibitor NPS2390, suggesting that CaSR mediates COM-induced OS. With regard to kidney stone formation in rats, crystals might cause renal injury and dysfunction. Pathological changes in the kidneys cause crystal retention. Lethal epithelial cell injury promotes crystal nucleation, aggregation, and retention. Sublethal injury or cell dysfunction may result in the generation of ineffective crystallization modulators and localized areas of supersaturation in the interstitium. Therefore, the increased CaOx-induced OS observed in the EG+GdCl_3_ group compared with the EG group could lead to increased crystal retention and dysfunction. Alternatively, the decreased OS detected in the EG+NPS2390 group might inhibit the vicious cycle of kidney stone formation.

The present study provides direct evidence that CaSR is present in HK-2 cells and rat kidneys. Notably, CaSR upregulation might increase intracellular MAPK signaling and induce OS, potentially leading to crystal retention and ultimately damage to renal function. These findings may provide an explanation for the role of CaSR in COM-induced renal injury.

## 5. Conclusion

In summary, activation of CaSR might lead to crystal retention and renal dysfunction by activation of MAPK pathways, as well as the production of excessive OS. These findings may be used to identify potential therapeutic and pharmacological targets.

## Figures and Tables

**Table 1:** Renal function and OS parameters in all groups ($\overset{-}{x}\pm s$).

Group	Serum BUN (mmol/L)	Serum Cr (*μ*mol/L)	MDA (nmol/mg protein)	SOD (*μ*/mg protein)	8-IP (pg/mL)
Control	8.45 ± 1.01	6.75 ± 0.84	1.18 ± 0.34	323.56 ± 35.93	2.96 ± 0.31
EG	10.62 ± 1.31	8.87 ± 0.89	1.99 ± 0.18^*∗*^	213.11 ± 15.37^*∗*^	8.11 ± 1.04^*∗*^
EG+GdCl_3_	15.54 ± 1.85^*∗*#^	17.16 ± 1.38^*∗*#^	3.56 ± 0.57^*∗*#^	138.26 ± 11.36^*∗*#^	17.87 ± 1.96^*∗*#^
EG+NPS2390	8.76 ± 0.52	7.74 ± 1.21	1.24 ± 0.11^#^	296.89 ± 31.94^#^	3.01 ± 0.63^#^

^*∗*^ *P* < 0.05 compared with the control group and ^#^ *P* < 0.05 compared with the EG group (*n* = 10).

## Abbreviations

CaSR: Calcium-sensing receptor
CaOx: Calcium oxalate
EG: Ethylene glycol
OS: Oxidative stress
SD: Sprague-Dawley
SOD: Superoxide dismutase
ROS: Reactive oxygen species
MDA: Malondialdehyde
MAPK: Mitogen-activated protein kinase
ERK: Extracellular signal-regulated protein kinase
JNK: c-Jun N-terminal protein kinase
OPN: Osteopontin
ESWL: Extracorporeal shock wave lithotripsy
URS: Ureteroscopy
PNL: Percutaneous nephrolithotomy
PBS: Phosphate-buffered saline
BUN: Blood urea nitrogen
Cr: Serum creatinine
GAPDH: Glyceraldehyde-3-phosphate dehydrogenase
SEM: Standard error of the mean
FBS: Fetal bovine serum
DMEM: Dulbecco's modified Eagle's medium.
